# Unravelling the hidden ancestry of American admixed populations

**DOI:** 10.1038/ncomms7596

**Published:** 2015-03-24

**Authors:** Francesco Montinaro, George B.J. Busby, Vincenzo L. Pascali, Simon Myers, Garrett Hellenthal, Cristian Capelli

**Affiliations:** 1 Institute of Legal Medicine, Catholic University, Largo F. Vito 1, Rome 00168, Italy; 2 Department of Zoology, University of Oxford, South Parks Road, Oxford OX1 3PS, UK; 3 Wellcome Trust Center for Human Genetics, Roosevelt Drive, Oxford OX3 7BN, UK; 4 Department of Statistics, University of Oxford, 1 South Parks Road, Oxford OX1 3TG, UK; 5 UCL Genetics Institute, University College London, WC1E 6BT Gower Street, UK

## Abstract

The movement of people into the Americas has brought different populations into contact, and contemporary American genomes are the product of a range of complex admixture events. Here we apply a haplotype-based ancestry identification approach to a large set of genome-wide SNP data from a variety of American, European and African populations to determine the contributions of different ancestral populations to the Americas. Our results provide a fine-scale characterization of the source populations, identify a series of novel, previously unreported contributions from Africa and Europe and highlight geohistorical structure in the ancestry of American admixed populations.

## Abstract (web summary)

The genetic make-up of people living in the Americas has been shaped heavily by migration. Here the authors use a haplotype-based approach to reconstruct American genomic ancestry using genotype data from 2,500 individuals, revealing a previously unrecognized genetic contribution from European and African populations.

## Graphical abstract

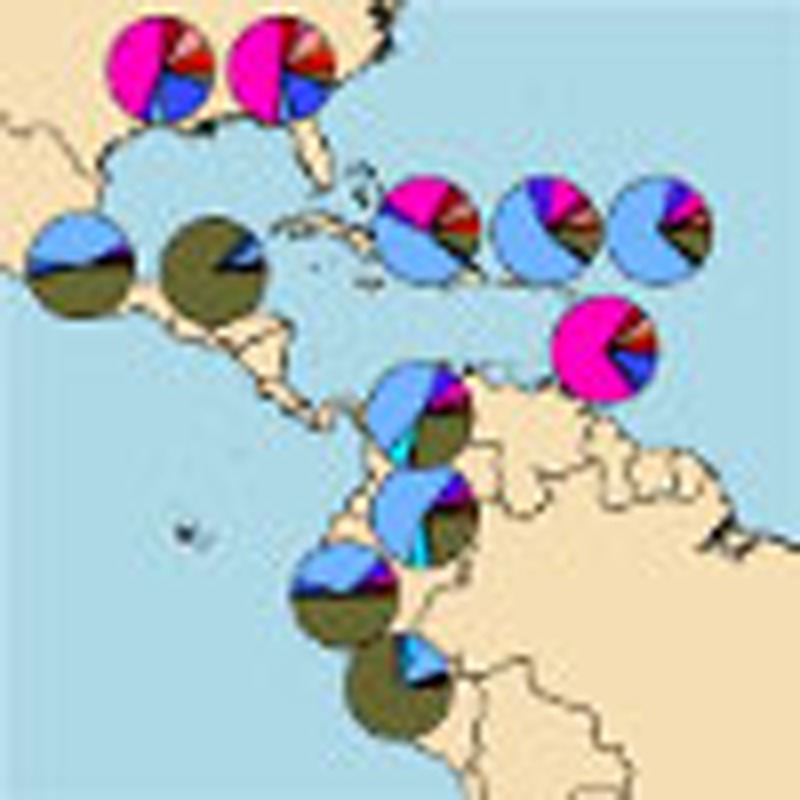

The genetic make-up of the Americas has been significantly shaped by the Colonial Era and the Atlantic slave trade. Given its historical and epidemiological implications, the estimation of the genetic ancestry of admixed American populations has been the subject of much attention^1^,^2^,^3^,^4^,^5^. However, despite historical evidence suggesting a wide heterogeneity in the European and African ancestry composition, sources have often been identified in terms of macrogeographic areas (for example, Southern versus Northern Europe) or by single populations as ‘consensus’ continental sources (for example, Yoruba from Nigeria for the whole of Africa). More recently, a significant contribution by the Spaniards has been highlighted for Caribbean and Southern American groups^4^,^5^. However, these methods, based on the local ancestry at a continental scale, make the identification of multiple sources from the same continent challenging.

In order to obtain a finer characterization of the ancestry landscape of admixed American populations, we implemented a novel inference method that reconstructs local genomic ancestry using a haplotype-based approach^6^,^7^. It has been shown in previous investigations^6^,^7^,^8^ that approaches based on haplotypes allow for a finer reconstruction of genetic structure when compared with classical approaches that directly employ single-marker genotypes, and that they are characterized by a lower degree of bias due to the ascertainment process of the polymorphisms studied^9^. We applied this methodology to genome-wide single-nucleotide polymorphisms (SNP) data from more than 2,500 individuals collected from various putatively admixed American and Caribbean populations. We compared the DNA of these ‘recipient’ groups to that of a cross-section of world-wide ‘donor’ populations that act as surrogates for the true ancestral source groups (Fig. 1, Supplementary Table 1), generating a detailed description of the genomic contribution of these groups to admixed American populations.

## Results

### Clustering of donor populations

**Figure 2:**
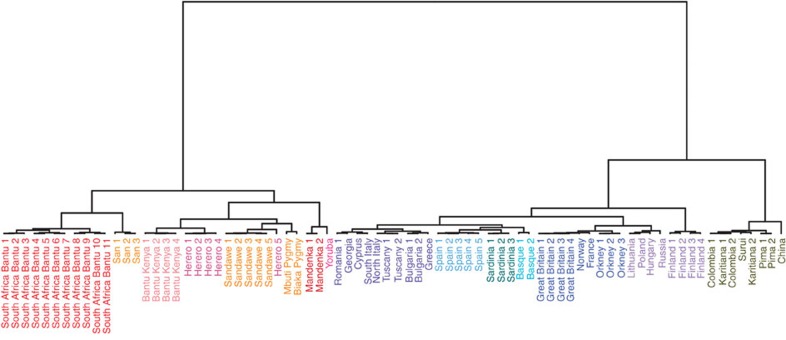
fineSTRUCTURE clustering of the analysed individuals. Tree of the analysed individuals pooled in 78 clusters as inferred by fineSTRUCTURE. Colours follow macro-area affiliations as in Fig. 1.

In order to minimize the impact of within-source genetic heterogeneity in the ancestry characterization process, we partitioned the 1,414 individuals from 42 population-label donors into genetically homogeneous clusters using a CHROMOPAINTER and fineSTRUCTURE analysis as described in the Methods section. This identified 78 clusters (Fig. 2, Supplementary Table 2) related by a hierarchical tree, with a broad correlation between clusters and geographic origin, allowing the grouping of clusters in 13 groups within Europe, Africa and East Asia/America (Fig. 2; Supplementary Table 2).

African individuals are divided within 33 clusters. Populations from West Africa showed a high degree of homogeneity, with all the Yoruba individuals from Nigeria forming a single cluster and the Mandenka from Senegal grouped into two. Individuals from Eastern and Southern Africa were distributed across 20 different clusters from three different regions (East Africa, South Africa and South West Africa), perhaps because of the complex demographic histories of populations from these areas^10^,^11^,^12^. In our collection of donor individuals, South-Central Africa is represented only by Bantu-speaking individuals from South Africa, while the South West Africa and the East Africa region clusters are represented exclusively by Herero and a Bantu speaker from South Africa (one individual from the HGDP data set^13^) and Bantu speakers from Kenya, respectively. Interestingly, one of the Herero individuals clusters together with Sandawe individuals instead of the other Herero individuals.

Pygmies, Sandawe and San (Khoisan/Pygmies^14^) were separated into clusters, essentially according to their population labels, although with some labelled groups differentiated into multiple clusters (Fig. 2, Supplementary Table 2).

European individuals are differentiated into 37 clusters that we grouped into six geographic regions (Fig. 2, Supplementary Table 2).

As previously reported, Sardinians and Basques formed population-specific groups^15^,^16^. Notably, by implementing the haplotype-based approach we were able to (i) detect eight individuals who are more related to the Basque population than to the Spanish individuals, within the Spanish data set included in the 1000 Genome Project panel (cluster ‘Basque 1’)^17^, probably reflecting a basque ancestry, and to (ii) differentiate them from the French Basque population included in the HGDP data set^13^ (‘cluster Basque 2’). We identified five Spanish clusters (‘SW Europe’; two of them including also a single French individual), highlighting the presence of a non-negligible heterogeneity in the country^18^.

The South-Eastern Europe group (‘SE Europe’) contains 10 clusters composed of individuals from Romania, Cyprus, Italy (excluding Sardinia), Bulgaria, Greece and France (one individual). Notably, Italian individuals are distributed into four different clusters according to their geographic origin (Supplementary Table 2).

A North-Western Europe group (‘NW Europe’) consists of eight clusters comprising individuals from British Isles, Orkney Islands, Norway, France, Germany and Austria. Similarly to the Basque populations, our approach clusters 23 individuals in a clade containing members of the Orcadian sample from the HGDP^13^.

The North-Eastern Europe group (‘NE-Europe’) is composed of eight clusters including individuals from Lithuania, Poland, Belarus, Hungary, Russia, Germany, Austria, Finland and Norway. Native American and East Asian (China) individuals are grouped into eight clusters, each exclusively containing individuals from the same labelled sample. These results confirm the extent of genetic structure in Africa and Europe, and provide a number of potential donor groups to the present-day American populations.

### Ancestry composition of the American populations

**Figure 3:**
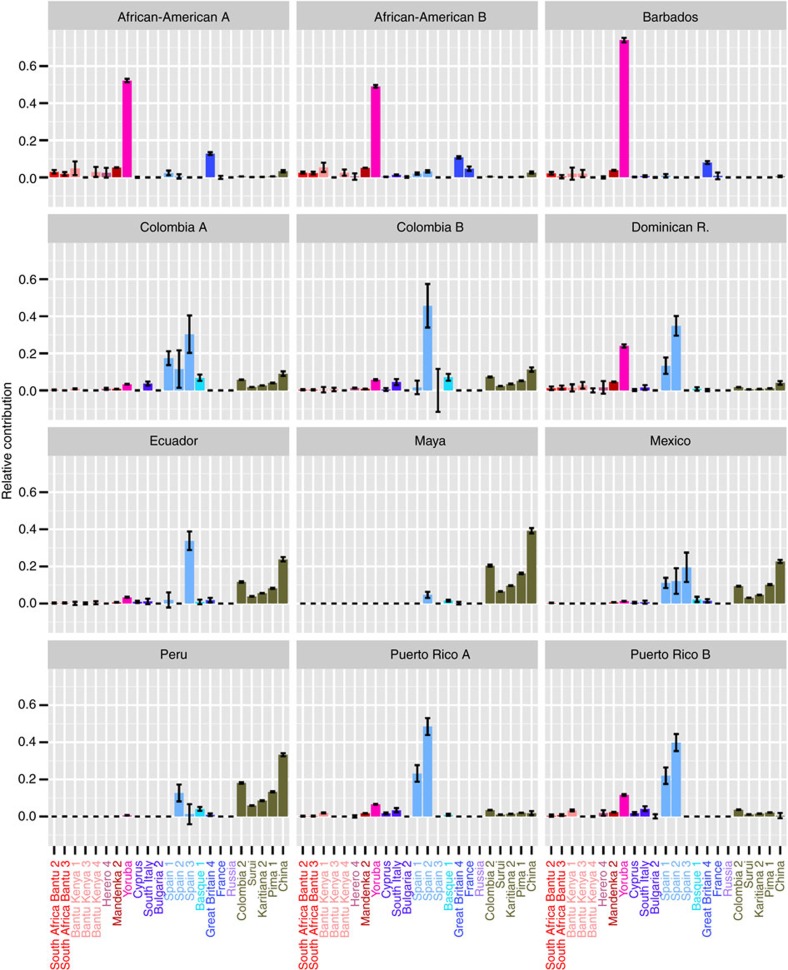
Contribution of the most by informative 23 clusters inferred by fineSTRUCTURE to the analysed recipient populations. Contribution of the most informative 23 clusters to the American and Caribbean populations estimated using the non-negative least square approach. Standard error based on jack-knife resampling (22 replicates) is reported.

We fit each of the American admixed populations as a mixture of the identified donor groups^19^ (see Methods, Supplementary Data 1). The contribution to the American admixed populations for the 23 most representative clusters and macro-areas is reported in Fig. 3 and Supplementary Fig. 1. This analysis assumes that haplotypes from the admixing populations are well represented within a mixture of present-day sampled groups. We were concerned that the demographic and evolutionary complexity of the peopling of the Americas^20^, coupled with the high genetic drift among Native American populations, might make the identification of the Native American contribution challenging. In particular, the true admixing groups from this region might be highly drifted from the possible ‘donor’ groups sampled, particularly given our geographically relatively sparse sample of such donor groups. To reduce this effect we always allowed a single well-sampled East Asian group (China) as a potential donor in the analysis, to act as a surrogate for haplotypes carried by any Native American donor population incompletely captured as a mixture of sampled Native American groups. Because this donor group is still likely to be strongly drifted relative to this East Asian ‘surrogate’, we also repeated our analysis after ‘masking’ direct copying of China population in the mixture-fitting step, although we still allowed all groups to contribute in the mixture. We compared the continental ancestry contributions from the full painting and the East Asian masked painting with an ADMIXTURE^21^ analysis performed at *K*=3 (Supplementary Figs 2 and 3), which closely matches the Africa, Europe and Asia/Native Americans partition. Continental ancestry estimates are highly correlated (*P* value <10^−12^) between all three approaches (Supplementary Fig. 2), although the squared distance between the masked continental ancestry estimates and that estimated by ADMIXTURE^21^ was, respectively, 5.4-fold and 7.9-fold reduced by the masking procedure for Europe and Asia/Native Americans, suggesting a slight gain in accuracy using this procedure. No major difference is seen for African contributions, while identified donor populations contributing to the mixture were very similar in both approaches; therefore, we henceforth report results on the basis of the masking procedure (Fig. 3).

Estimated African ancestry ranges from virtually 0 (Maya) to 0.87 (Barbados) in all the analysed populations.

Caribbean populations show a higher African component than Southern American ones, consistent with historical records that documented a larger number of slaves in the Caribbean Islands^22^,^23^.

**Figure 4:**
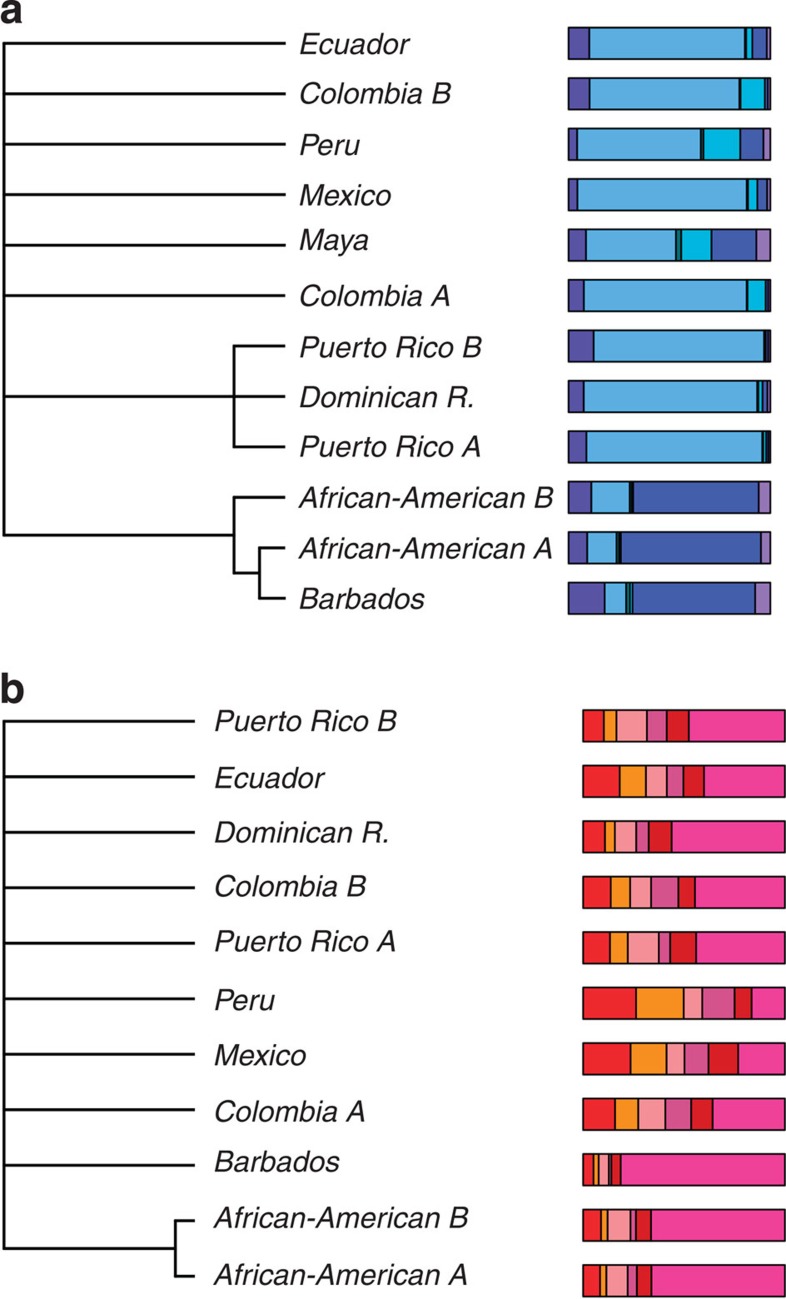
Hierarchical consensus trees of the continental components for American and Caribbean populations. Consensus tree using Hierarchical clustering for (**a**) European component; (**b**) African component. Bar plots at the tips of the trees indicate the relative ancestry composition of the analysed population; colours refer to the 13 groups in Fig. 1. Only branches supported by more than 80% of the 1,000 trees built by bootstrap described in Methods are retained.

Although our sampling of Africans is incomplete, we see variation among groups in similarity to present-day populations from different parts of Africa. In all groups, the Yorubans from West Africa are the largest contributor, confirming this region as the major component of African slaves^1^,^2^,^4^. However, our fine-scale analysis suggests additional genetic contributions from populations from other parts of Africa, with contributions from particular groups sampled in Senegambia (the Mandenka), Southern (South African Bantu language speakers) and Eastern Africa (Kenyan Bantu language speakers) identified in 6 out of 12 populations we investigated. Historical reports indicate that Senegambia and South-Eastern Africa contributed an average of 6 and 4% of all disembarked slaves to the Americas (totalling several hundreds of thousands individuals), respectively, with ethnic groups from Senegal and Mozambique being among the 10 most prominent according to slavery documentation^22^. In addition, more than 30% of the total slaves arriving in mainland Spanish America up to the 1630s came from Senegambia^23^, and we accordingly find that the relative contribution from the Mandenka is higher in all areas historically under the Spanish rule (Fig. 4).

The degree of resolution in the identification of the sources provided by our approach is also evident in the fine characterization of the European component, which ranges between 0.078 (Barbados) and 0.79 (Puerto Rico). We specifically identify Spaniards among other available Southern European populations as the most represented European source for all nine Hispanic/Latino populations. In contrast, the most represented European sources in the Afro-Americans and Barbadians were Great Britain clusters (Figs 3 and 4a), in full agreement with historical records^24^,^25^; a small amount of Spanish ancestry is also inferred in these groups. Interestingly among the Spaniards, two clusters do not contribute to any of the analysed populations, presumably reflecting a differential contribute of Iberian regions to the genetic pool of American populations.

Among smaller genetic contributions, we identify for the first time a genetic signature of Basque ancestry in five (out of six) of the Continental South American populations, ranging between 0.015 in the Maya population to 0.07 in Colombia. It has been documented that Basque individuals were a considerable fraction of Spanish immigrants in the XVI and XVII centuries, especially to Mexico, Cuba, Chile, Peru and Colombia^26^. These results could explain, at least in part, the recently observed structure in the Spanish component of the Continental but not Caribbean populations^4^.

Among the remaining European clusters the most represented, contributing to five of the analysed populations, is composed of individuals from South Italy and Sicily. This might indicate a minor contribution from the Italian peninsula as documented in historical records^27^. Interestingly, we also identified a considerable fraction of French ancestry in one African-American sample, in agreement with French immigration into the Southern United States during colonial times^28^,^29^.

At the individual level, the analysis highlights a high heterogeneity in several analysed populations (Supplementary Fig. 4), as expected given recent admixture. This is particularly evident in the African-American populations, in which, for the African ancestry, the inferred contributions of Mandenka and W Africa range from 0 to 35% and 0 to 100%, respectively. For the European contribution, a few individuals possessed a high degree of inferred Spanish (95% confidence interval (CI) 0–0.27) or Italian ancestry (95% CI 0–0.14), while global Native American ancestry varies from 0 to 65%.

### Clusters versus population-label-based ancestry reconstruction

We explored the variation in ancestry determination when using a population-label-based approach instead of a clustering-based one by comparing estimates obtained using the same set of source individuals but grouped in different ways (Supplementary Fig. 5). Population labels might mask contributions, by for example, falsely grouping genetically distinct donor populations with different actual contributions to an admixed population. In accordance with this concern, although results were mainly similar, the label-based approach inferred the French population (partially replacing Great Britain) as the major source for the African-American and Barbados samples and no longer detected the Basques as a source population. A more refined ancestry depiction by a cluster-based approach is not unexpected for the European sources, given the population stratification following the complex ancient and more recent admixture history of the continent^7^,^13^,^30^,^31^. These results indicate that using fine-scale genetics-based clustering methods on the basis of phased data to replace or supplement sample-based labels can strongly improve the resolution of ancestry reconstruction.

### Analysis of relative ancestry composition

We used a hierarchical clustering algorithm on the basis of the Euclidean distances between relative ancestry proportions to explore the dissimilarities in source composition across admixed populations (Fig. 4) and constructed the 80% consensus tree of 1,000 simulated data sets (see Methods section).

Clustering based on European components broadly support two groups of recipient populations: one containing Afro-Americans and Barbadians, the other containing all of the remaining populations (Fig. 4a). Notably, these clusters match the English and Spanish colonies in the Americas and reflect geohistorical differences in the migration pattern from the Northern hemisphere^23^ (Voyages: The Trans-Atlantic Slave Trade Database: http://www.slavevoyages.org/tast/assessment/estimates.faces) as suggested by their different European source composition (Fig. 4a). In addition, the Caribbean Islands Puerto Rico and Dominican Republic tend to cluster together, probably reflecting a different migration pattern between Caribbean and mainland America.

On the other end no particular clustering, apart from between the two African-American groups, emerges when the African relative composition is considered, reflecting the complexity of the slave trade dynamics (Fig. 4b).

## Discussion

Our results provided new insights into the genetic make-up of American populations, highlighting the underappreciated heterogeneity of ancestral components across American populations and the power of haplotype-based analytical techniques in identifying fine-scale ancestry without strong prior assumptions. The application of this approach to additional admixed populations (for example, Brazilians) and the inclusion of more sources, particularly from Africa and the Americas, are expected to further clarify the complexity of the ancestry composition of the American continent.

## Methods

### Data set

We assembled from literature a data set composed of 4,139 individuals from 64 populations sampled from Europe, Africa, East Asia (represented by a single sample from China) and the Americas, genotyped with different Illumina platforms (Supplementary Table 1). The data set was filtered using PLINK ver. 1.07 (ref. 32) to retain only SNPs and individuals with genotyping success rate >98%, retaining 250,800 autosomal markers.

We screened the pruned data set using KING^33^ to remove individuals with kinship parameter higher than 0.0884 as potentially related as indicated in the software’s manual. The final data set is composed of 3,960 individuals from 64 populations. Of these, 12 were treated as ‘recipients’ (African-American A, African-American B, Barbados, Colombia A, Colombia B, Dominican Republic, Ecuador, Maya, Mexico, Peru, Puerto Rico A and Puerto Rico B), and the remaining 52 as donors, as described below.

### Phasing

The data set was phased using the Segmented Haplotype Estimation and Imputation tool ver. 2 (ShapeIT) software^34^, which improves the Hidden Markov model implemented in IMPUTE2 (ref. 35) and MaCH^36^ by increasing the speed and accuracy of the phasing process. We used the HapMap^37^ human genome build 37 recombination map downloaded from the ShapeIT website ( https://mathgen.stats.ox.ac.uk/genetics_software/shapeit/shapeit.html#gmap).

### Clustering of donor populations

As a first step, we clustered the individuals belonging to ‘donor’ populations into homogenous groups. This approach allows a more detailed reconstruction of the ancestry of a given population, taking into account the genetic structure of the donor demes.

First, we used a novel inferential algorithm implemented in CHROMOPAINTER^6^ to obtain the most relevant genealogical information about the local ancestry of analysed individuals. The algorithm uses a modification of the Hidden Markov Model proposed in ref. 38, which reconstructs (also referred as ‘paints’) an individual’s chromosomes as a series of genomic fragments from potential donor individuals, using the information on the allelic state of recipient and donors at each available position along the chromosome. In practice, we ‘painted’ the genomic profile of each donor individual as the combination of fragments received from other donor chromosomes. We used a value of 267 for the ‘recombination scaling constant’ (which controls the average switch rate of the HMM) Ne, and 0.00043 for the ‘per site mutation rate’ Θ, nuisance parameters, as estimated by 10 iterations of the expectation-maximization algorithm in CHROMOPAINTER. This algorithm finds the local optimum values of these parameters iterating over the data. Given the computational complexity of this process, the estimation of these two parameters was obtained averaging the values calculated from an analysis performed on a subset of six representative populations (Luhya, Yoruba B, Tuscany B, Great Britain, Karitiana and Pima) and five randomly selected chromosomes (2, 5, 8, 16 and 22).

Second, we analysed the painted data set using fineSTRUCTURE^6^, in order to identify homogenous clusters. In detail, the inference of population assignment is performed through a Markov chain Monte Carlo (MCMC) algorithm related to that implemented in the STRUCTURAMA software^39^, while the number of clusters is inferred using a RJ-MCMC algorithm that proposes new configurations from the previous step and is accepted with a probability depending on the ratio between the two respective Likelihoods.

We analysed CHROMOPAINTER's output performing two different MCMC runs, each composed by 5 million iterations, and extracted the Maximum *A Posteriori* state characterized by the higher likelihood.

### Painting of the recipient populations

We painted each individual belonging to recipient populations as a combination of genomic fragments inherited by ‘donor individuals’ pooled using the clustering affiliation obtained as previously described and summarized in Supplementary Table 2. We used the same inferred values of Ne and Θ from the previous section to do so. In this analysis, the average number of SNPs across all haplotype segments painted contiguously using a single donor individual was ~17 SNPs (95% CI: 13–32).

### Ancestry assignment

CHROMOPAINTER provides a digested output of the reconstructed individual’s chromosomes in the form of a ‘copying vector’, which is a summary of the amount of DNA copied genome-wide from each donor population. By normalizing this vector to sum to 1, it is possible to obtain a representation of the proportion of genome copied from each donor population by each recipient individual. We identified the most closely ancestrally related donor population for each Afro-American and Latino/Hispanic population by comparing their copying vectors to copying vectors inferred in the same way for each of the donor clusters, using the non-negative least square function^19^ in R 2.14. Briefly, this approach identifies copying vectors of donor populations that better match the copying vector of recipient populations as estimated by CHROMOPAINTER. For each recipient population, we decomposed the ancestry of that group as a mixture (with proportions summing to 1) of each sampled potential donor cluster, by comparing the ‘copying vector’ donor and recipient populations. In addition to controlling for variation in sample size across our donor groups, this approach also accounts for the fact that human populations are genetically related, and so most haplotypes are shared, exploiting subtle signals relating to average copying probabilities to distinguish among often closely related potential donor groups. Note, however, that if true donor groups are not sampled, they cannot be included, and in this setting the method is likely to instead choose the ‘closest’ among the sampled groups. Therefore, the groups identified using the approach should be considered as the most ancestrally related populations.

In order to avoid any possible distortion in the assignment, we removed all the clusters composed only by a single individual.

In addition, given prior knowledge of strong genetic bottlenecks that have shaped the gene pool of modern Native American populations, we anticipate extremely strong genetic drift of these specific admixing groups, relative to East Asian groups with whom they still share ancestral haplotypes. Because the mixture decomposition does not model such drift, which is expected to be reflected in inaccurately modelled (that is, over-estimated) copying from the ‘East Asia’ group in particular, we re-performed the mixture analysis, removing the contribution that each population copied from China, in order to ameliorate the impact of such recent drift.

The ancestry composition of each individual within the recipient populations was estimated using the same approach as described above but comparing the individual’s copying vector to the source population-copying vector. Results are reported in Supplementary Fig. 4.

The uncertainty in the ancestry estimation at the population level was assessed by applying a jack-knife approach, and estimating the s.e. as in ref. 40 (Fig. 3).

In addition, for comparative purposes we performed the same analysis using generally coarser population labels, instead of clusters inferred by fineSTRUCTURE (Supplementary Fig. 5).

### African and European relative contributions

The relative African and European ancestry composition was calculated using the results described above and reported in Fig. 3, and Supplementary Data 1, normalized to 1 by grouping sources according to their continental origin (Fig. 4). The degree of clustering for the relative continental ancestry contribution was explored by hierarchical cluster performed using the ‘ward’ method on the Euclidean distance matrix (Fig. 4). Given the low amount of African ancestry in Maya individuals, we excluded this population from this analysis.

We built a consensus tree (retaining only branches with >80% support) based on 1,000 bootstrapped simulated samples, using the ‘ape’ R package^41^. In detail, we simulated 1,000 populations of *n* individuals, where *n* is the size of each analysed sample. Each individual was generated by combining 22 ‘painted’ chromosomes randomly selected from the analysed population.

## Additional information

**How to cite this article:** Montinaro, F. *et al*. Unravelling the hidden ancestry of American admixed populations. *Nat. Commun*. 6:6596 doi: 10.1038/ncomms7596 (2015).

## Supplementary Material

Supplementary Figures, Tables and References Supplementary Figures 1-5, Supplementary Tables 1-2 and Supplementary References

Supplementary Data 1 Ancestry composition for recipient populations; standard error estimated by 22 jack-knife resampling indicated in brackets.

## Figures and Tables

**Figure 1:**
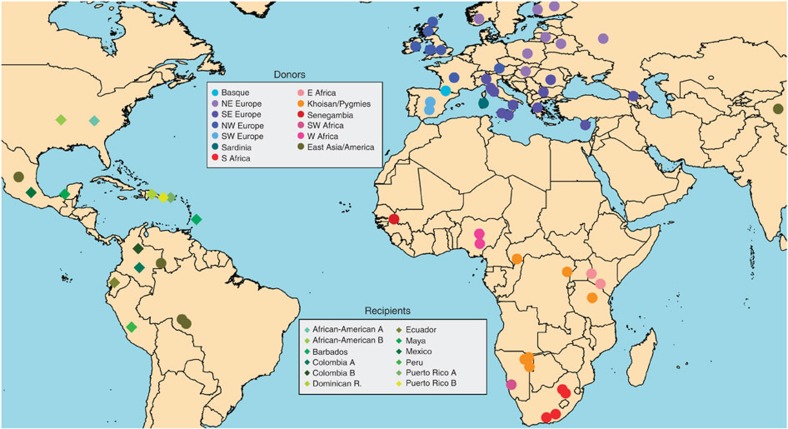
Approximate geographic sampling location of donor and recipient populations analysed. Colours refer to the 13 groups as described in Fig. 2 and Supplementary Table 2. Circles and diamond refer, respectively, to donors and recipients.
