# Supplementary material for: Unravelling the hidden ancestry of American admixed populations
Source: Nat Commun. 2015 Mar 24;6:6596. doi: 10.1038/ncomms7596 (PMC4374169; doi:10.1038/ncomms7596)
Supplement: Supplementary Figures, Tables and References — Supplementary Figures 1-5, Supplementary Tables 1-2 and Supplementary References [file ncomms7596-s1.pdf]

Supplementary Information

Unravelling the hidden ancestry of American admixed populations.

Francesco Montinaro[1,2], George B.J. Busby[2,4], Vincenzo L. Pascali[1], Simon Myers[3,4], Garrett Hellenthal[5] and Cristian Capelli[2*].

[1] Institute of Legal Medicine, Catholic University, Largo F. Vito, 1-00168, Rome, Italy

[2] Department of Zoology, University of Oxford, South Parks Road, Oxford, OX1 3PS, United Kingdom

[3] Department of Statistics, University of Oxford,1 South Parks Road, , OX1 3TG, Oxford, United Kingdom

[4] Wellcome Trust Center for Human Genetics, Roosevelt Drive, OX3 7BN Oxford, United Kingdom

[5] UCL Genetics Institute, University College London, WC1E 6BT, Gower Street,United Kingdom

*Corresponding author: Dr. Cristian Capelli: cristian.capelli@zoo.ox.ac.uk

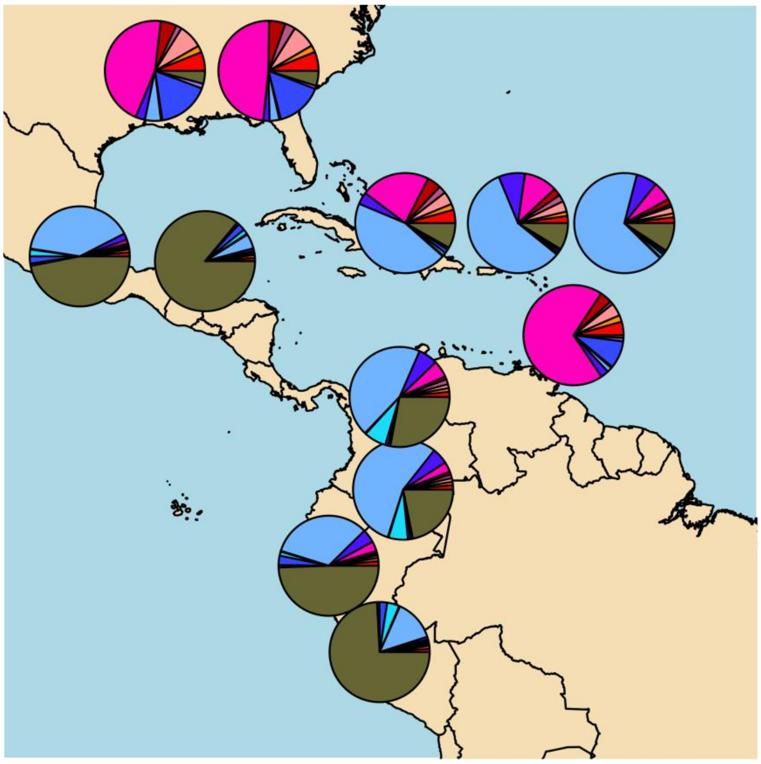

Supplementary Fig. 1: Ancestry composition of recipient populations based on the contribution of the 13 macro-areas identified by the means of fineSTRUCTURE analysis. Colors refer to the groups in Fig 1 and Fig. 2

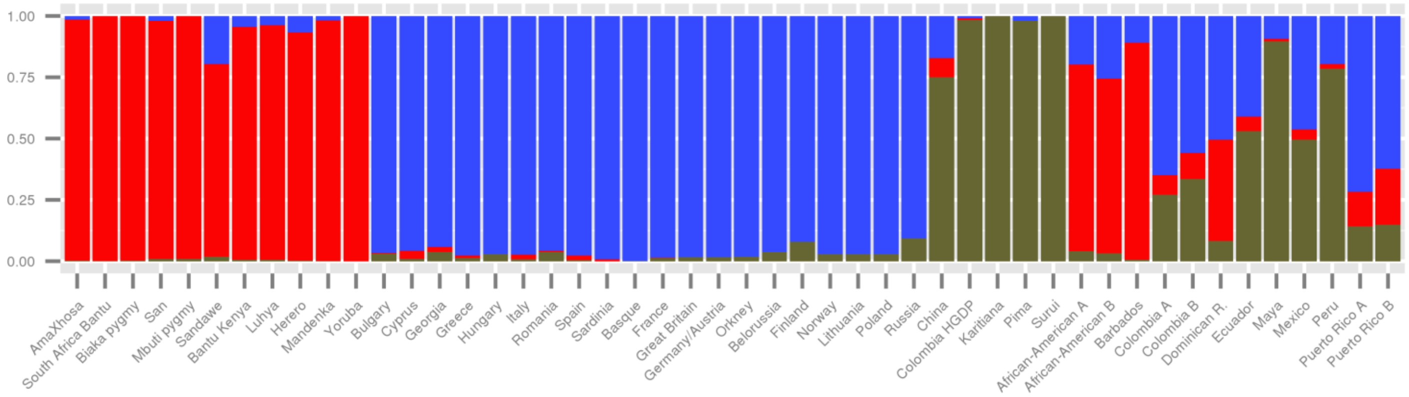

Supplementary Fig. 2: Average population ancestry estimated by ADMIXTURE analysis for the analyzed populations. The sample size of each population is reported in Supplementary Table 1.

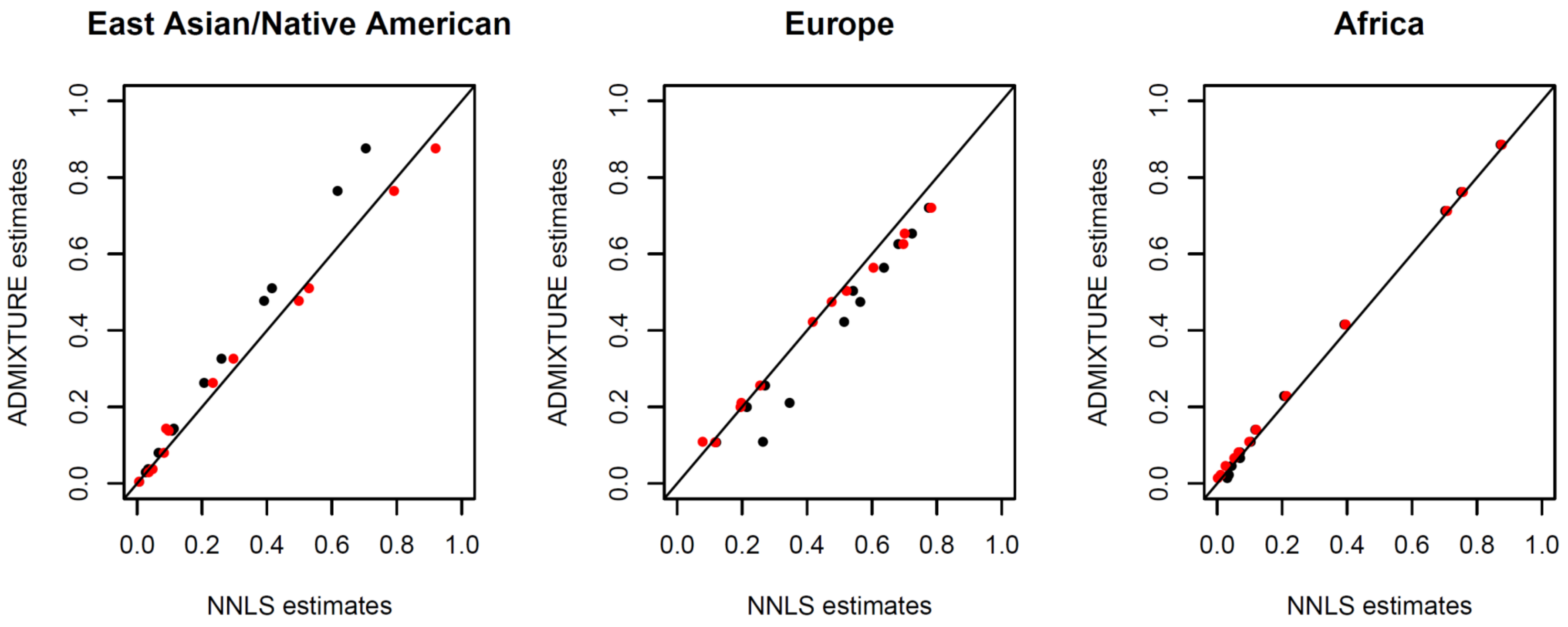

Supplementary Fig. 3: Comparison between East Asian “masked” and “unmasked” ancestry estimations vs. ADMIXTURE analysis. Each plot shows the relationship between the Continental ancestry estimated by ADMIXTURE at K=3 and that obtained by NNLS masking (red dots) or maintaining (black dots) East Asian contribution.

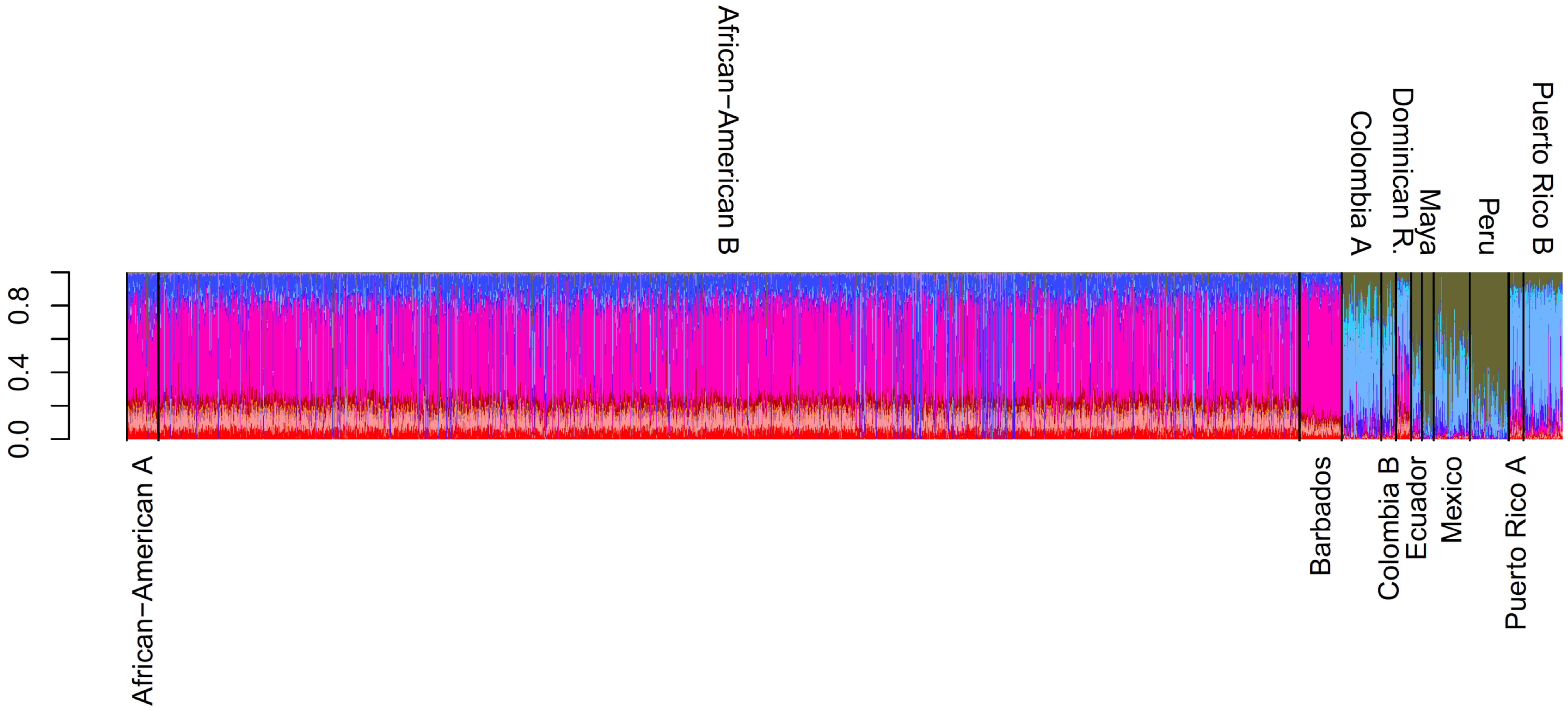

Supplementary Fig. 4: Individual ancestry composition estimated using the NNLS approach (see Methods) for the recipient populations analyzed. Each bar plot represents an individual and the colors follow group affiliation as in Fig 1 and Supplementary Table 1.

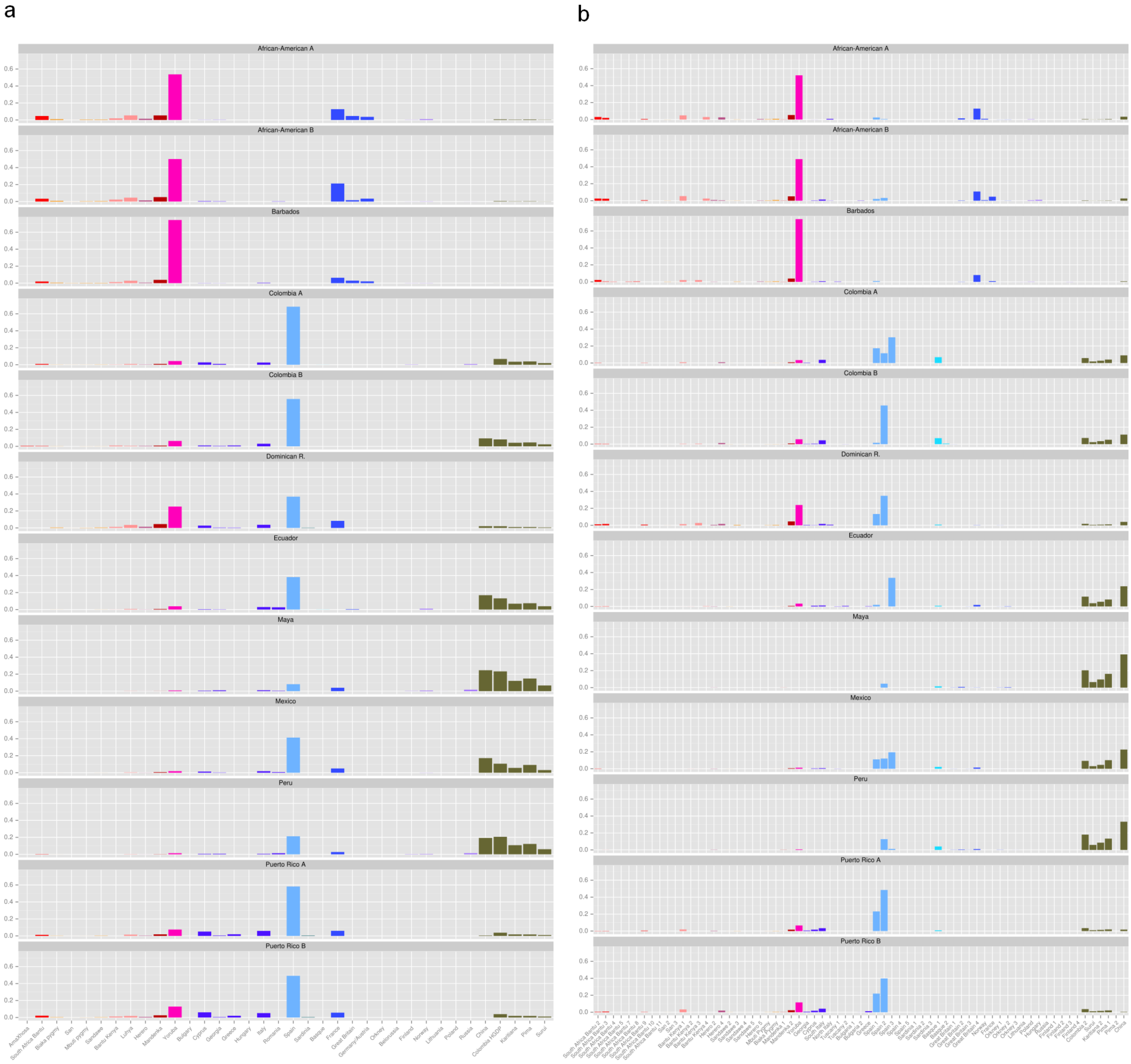

Supplementary Fig. 5: Comparison between population and cluster based ancestry estimates. Ancestry estimates using (a) populations or (b.) clusters as inferred by fineSTRUCTURE.

Africa	Group		Pre QC	After QC	Ref.	Europe	Macro-Area		Pre QC	After QC	Ref.
	W Africa						*SE Europe*				
		Yoruba A	101	101	*(1)*			Spain A	97	97	*(1)*
		Yoruba B	21	21	*(2)*			Spain B	34	34	*(7, 8)*
	Senegambia										
		Mandenka	22	22	*(2)*		*SW Europe*				
	E Africa							Bulgary	18	18	*(7)*
		Bantu Kenya	11	10	*(2)*			Cyprus	12	12	*(8)*
		Luhya	97	87	*(1)*			East Sicily	10	10	*(7)*
	SW Africa							North Italy	12	12	*(2)*
		Herero	12	12	*(3)*			Georgia	20	20	*(9)*
	S Africa							Greece	20	20	*(7)*
		AmaXhosa	15	15	*(4)*			Romania	14	14	*(8)*
		South Africa Bantu A	8	6	*(2)*			Tuscany A	100	98	*(1)*
		South Africa Bantu B	20	20	*(3)*			Tuscany B	8	8	*(2)*
		South Africa Bantu C	94	94	*(5)*			South Italy	18	18	*(7)*
	*Khoisan/Pygmies**							West Sicily	10	10	*(7)*
		Biaka pygmy	21	21	*(2)*						
		Mbuti pygmy	13	13	*(2)*		*Basque*				
		San A	19	19	*(3)*			Basque	24	24	*(2)*
		San B	18	15	*(4)*						
		San C	5	5	*(2)*		*Sardinia*				
		Sandawe	28	23	*(6)*			Sardinia	28	28	*(2)*
**Europe**						**East Asia/America**					
	NW Europe							Colombia	7	7	*(2)*
		England	6	6	*(7)*			Karitiana	14	14	*(2)*
		France	28	28	*(2)*			Pima	14	14	*(2)*
		Great Britain	99	96	*(1)*			Surui	8	8	*(2)*
		Ireland	7	7	*(7)*			China	100	100	*(1)*
		Orkney	15	15	*(2)*						
		Norway	18	18	*(7)*						
		Scotland	6	6	*(7)*						
		Wales	4	4	*(7)*						
	NE Europe					**Recipients**					
		Finland A	100	100	*(1)*			African-American A	62	56	*(1)*
		Finland B	2	2	*(7)*			African-American B	2162	2023	*(10)*
		Germany and Austria	4	4	*(7)*			Barbados	75	75	*(1)*
		Lithuania	10	10	*(8)*			Colombia A	70	70	*(1)*
		Hungary	20	19	*(8)*			Colombia B	26	26	*(11)*
		Poland	16	16	*(7)*			Dominican Republic	27	27	*(11)*
		Belorussia	8	8	*(8)*			Ecuador	20	19	*(11)*
		Russia	25	25	*(2)*			Maya	21	21	*(2)*
								Mexico	68	64	*(1)*
								Peru	70	69	*(1)*
								Puerto Rico A	70	70	*(1)*
								Puerto Rico B	27	26	*(11)*
						**TOTAL**			**4139**	**3960**	

*The term “Khoisan” refers to Guldemann and Fehn, 2014[12].

Supplementary Table 1: Overview of the analyzed populations and their sample size before (pre QC) and after (After QC) the quality control described in the Methods section.

Continent	Group	Cluster Name	Cluster composition
**Africa**			
	W Africa		
		Yoruba	Yoruba A 101;Yoruba B 21
	Senegambia		
		Mandenka 1	Mandenka 3
		Mandenka 2	Mandenka 19
	E Africa		
		Bantu Kenya 1	Bantu Kenya 2
		Bantu Kenya 2	Luhya 2
		Bantu Kenya 3	Luhya 39;Bantu Kenya 6
		Bantu Kenya 4	Luhya 46;Bantu Kenya 2
	SW Africa		
		Herero 1	Herero 1
		Herero 2	Herero 6
		Herero 3	Herero 1
		Herero 4	South Africa Bantu A 1;Herero 1
		Herero 5	Herero 3
	S Africa		
		South Africa Bantu 1	South Africa Bantu B 1
		South Africa Bantu 2	South Africa Bantu C 8; Amaxhosa 1
		South Africa Bantu 3	South Africa Bantu C 8
		South Africa Bantu 4	South Africa Bantu C 7
		South Africa Bantu 5	South Africa Bantu C 6
		South Africa Bantu 6	South Africa Bantu B 1
		South Africa Bantu 7	South Africa Bantu C 2
		South Africa Bantu 8	South Africa Bantu C 9;AmaXhosa 13; South Africa Bantu B 2
		South Africa Bantu 9	South Africa Bantu C 13;South Africa Bantu A 3;South Africa Bantu B 6
		South Africa Bantu 10	South Africa Bantu C 18;South Africa Bantu A 1;South Africa Bantu B 3
		South Africa Bantu 11	South Africa Bantu C 23;South Africa Bantu B 7;AmaXhosa 1 South Africa Bantu A 1
	Khoisan/Pygmies		
		San 1	San B 1
		San 2	San B 7;San A 5;San C 2
		San 3	San B 7;San A;14 San C 3
		Sandawe 1	Sandawe 1
		Sandawe 2	Sandawe 2
		Sandawe 3	Sandawe 8
		Sandawe 4	Sandawe 3
		Sandawe 5	Sandawe 9
		Mbuti Pigmy	Mbuti Pigmy 13
		Biaka Pigmy	Biaka Pigmy 21
**Europe**			
	NW Europe		
		Great Britain 1	Great Britain 2
		Great Britain 2	Great Britain 29
		Great Britain 3	Great Britain 2
		Great Britain 4	Great Britain 40;Ireland 7;Scotland 6;France 3;England 6;Wales 4; Germany and Austria 2
		Norway	Norway 17
		France	France 22
		Orkney 1	Great Britain 2
		Orkney 2	Great Britain 18;Orkney 6
		Orkney 3	Great Britain 3, Orkney 3
	NE Europe		
		Lithuania	Lithuania 9
		Poland	Poland 16;Belorussia 8; Lithuania 1
		Hungary	Hungary 19;Germany and Austria 2
		Russia	Russia 25
		Finland 1	Finland A 20

	Finland 2	Finland A 44,Finland B1
	Finland 3	Finland A 7, Norw ay 1
	Finland 4	Finland A 29, Finland B 1
SW Europe		
	Spain 1	Spain A 5
	Spain 2	Spain A27;Spain B 12
	Spain 3	Spain A24;Spain B 13
	Spain 4	Spain A 18;Spain B 7;France 1
	Spain 5	Spain A 15;Spain B 2;France 1
SE Europe		
	Romania 1	Romania 1
	Cyprus	Cyprus 12
	South Italy	West Sicily 9;South Italy 18; East Sicily10
	Georgia	Georgia 19
	Bulgaria 1	Bulgaria 2;Georgia 1
	Bulgaria 2	Romania 13;Bulgary 16
	Greece	Greece 20, West Sicily 1
	North Italy	France 1;North Italy 12
	Tuscany 1	Tuscany A 61;Tuscany B 8
	Tuscany 2	Tuscany A 37
Basque		
	Basque 1	Spain A 8
	Basque 2	Basque 24
Sardinia		
	Sardinia 1	Sardinia 6
	Sardinia 2	Sardinia 9
	Sardinia 3	Sardinia 13
**East Asia/America**		
	Karitiana 1	Karitiana 1
	Colombia 1	Colombia 1
	Colombia 2	Colombia 6
	Surui	Surui 8
	Karitiana 2	Karitiana 13
	Pima 1	Pima 7
	Pima 2	Pima 7
	China	China 100

Supplementary Table 2: Overview and composition of the clusters inferred by fineSTRUCTURE analysis.

Supplementary References:

1. Consortium, T. 1000 G. P. A map of human genome variation from population-scale sequencing. *Nature* **467,** 1061–1073 (2010).
2. Li, J. Z. *et al.* Worldwide human relationships inferred from genome-wide patterns of variation. *Science* **319,** 1100–1104 (2008).
3. Schlebusch, C. M. *et al.* Genomic Variation in Seven Khoe-San Groups Reveals Adaptation and Complex African History. *Science* **338,** 374–379 (2012).
4. Petersen, D. C. *et al.* Complex Patterns of Genomic Admixture within Southern Africa. *PLoS Genet* **9,** e1003309 (2013).
5. May, A. *et al.* Genetic diversity in black South Africans from Soweto. *BMC Genomics* **14,** 644 (2013).
6. Henn, B. M. *et al.* Hunter-gatherer genomic diversity suggests a southern African origin for modern humans. *PNAS* **108,** 5154–5162 (2011).
7. Hellenthal, G. *et al.* A Genetic Atlas of Human Admixture History. *Science* **343,** 747–751 (2014).
8. Behar, D. M. *et al.* The genome-wide structure of the Jewish people. *Nature* **466,** 238–242 (2010).
9. Yunusbayev, B. *et al.* The Caucasus as an asymmetric semipermeable barrier to ancient human migrations. *Mol Biol Evol* msr221 (2011). doi:10.1093/molbev/msr221
10. Illumina Inc. Illumina IControl Database. at <http://genomeinformaticsalliance.org/science/icontroldb.ilmn>
11. Bryc, K. *et al.* Genome-wide patterns of population structure and admixture among Hispanic/Latino populations. *Proceedings of the National Academy of Sciences* **107,** 8954–8961 (2010).
12. Güldemann, T. & Fehn, A.-M. *Beyond 'Khoisan': Historical relations in the Kalahari Basin*. (John Benjamins Publishing Company, 2014).
